# Complementary, Cooperative Ditopic Halogen Bonding and Electron Donor-Acceptor π-π Complexation in the Formation of Cocrystals

**DOI:** 10.3390/molecules27051527

**Published:** 2022-02-24

**Authors:** Erin D. Speetzen, Chideraa I. Nwachukwu, Nathan P. Bowling, Eric Bosch

**Affiliations:** 1 Department of Chemistry, University of Wisconsin-Stevens Point, 2101 South Avenue, Stevens Point, WI 54481, USA; espeetze@uwsp.edu (E.D.S.); nbowling@uwsp.edu (N.P.B.); 2 Chemistry Department, Missouri State University, 901 South National Avenue, Springfield, MO 65897, USA; nwachukwu123@live.missouristate.edu

**Keywords:** ditopic halogen bonding, electron donor acceptor complexation, pi-stacking, cooperative halogen bonding and electron donor acceptor complexation, complementary halogen bonding and electron donor acceptor complexation, charge-transfer complexation, tetrel bonding

## Abstract

This study expands and combines concepts from two of our earlier studies. One study reported the complementary halogen bonding and π-π charge transfer complexation observed between isomeric electron rich 4-*N*,*N*-dimethylaminophenylethynylpyridines and the electron poor halogen bond donor, 1-(3,5-dinitrophenylethynyl)-2,3,5,6-tetrafluoro-4-iodobenzene while the second study elaborated the ditopic halogen bonding of activated pyrimidines. Leveraging our understanding on the combination of these non-covalent interactions, we describe cocrystallization featuring ditopic halogen bonding and π-stacking. Specifically, red cocrystals are formed between the ditopic electron poor halogen bond donor 1-(3,5-dinitrophenylethynyl)-2,4,6-triflouro-3,5-diiodobenzene and each of electron rich pyrimidines 2- and 5-(4-*N*,*N*-dimethyl-aminophenylethynyl)pyrimidine. The X-ray single crystal structures of these cocrystals are described in terms of halogen bonding and electron donor-acceptor π-complexation. Computations confirm that the donor-acceptor π-stacking interactions are consistently stronger than the halogen bonding interactions and that there is cooperativity between π-stacking and halogen bonding in the crystals.

## 1. Introduction

**Figure 1:**
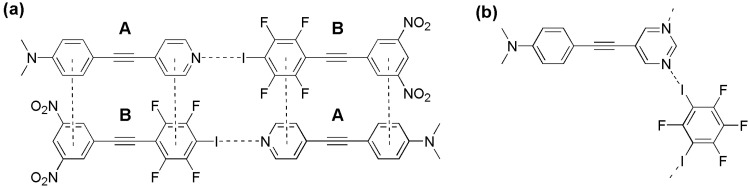
(**a**) Cooperative halogen bonding and EDA π-stacking in cocrystal **A•B** [12]. (**b**) Ditopic halogen bonding in cocrystals formed between 5-(*N*,*N*-dimethylaminophenylethynylpyrimidine and 1,3-diiodotetrafluorobenzene [13].

The cooperative interplay between non-covalent interactions is a current topic of interest [1,2]. Indeed, the deliberate preparation of supramolecular complexes, polymers and networks in the crystalline state relies on the intentional exploitation of multiple cooperative non-covalent interactions [3,4,5,6,7]. While it is reasonable to expect concomitant π-stacking in halogen bonded cocrystals formed between halobenzenes and pyridines the intentional coupling of halogen bonding and π-stacking is increasingly recognized as important in crystal engineering and supramolecular assembly. Halogen bonding and π-stacking have been incorporated as key cooperative non-covalent interactions in the [2+2] photoreaction in oocrystals including dipyridylethylenes [8]. Cooperative halogen bonding and π-stacking also feature in the preparation of luminescent materials [9] and semiconductors [10] amongst other applications. Clearly, recognition and exploitation of charge-transfer in p-stacked interactions has potential in optoelectronics [11]. Earlier we described cocrystallization based on cooperative halogen bonding and electron donor-electron acceptor π-complexation to form colored cocrystals [12]. In that study, which forms the basis for the current report, two complimentary molecules were designed to each include a halogen bonding site and an enhanced electron donor-acceptor π-complexation as illustrated in Figure 1a. Thus, molecule A has a pyridyl moiety as halogen bond acceptor along with an electron rich *N*,*N*-dimethylaminophenyl moiety for enhanced electron donor-acceptor (EDA) π-stacking. Complementary molecule B has an iodotetrafluorophenyl moiety as halogen bond donor and an electron poor dinitrophenyl moiety for complementary EDA π-stacking. Slow evaporation of an equimolar solution of A and B yielded a homogeneous mass of bright red crystals. The single crystal X-ray structure of the red crystals confirmed the formation of alternating -ABAB- π-stacked, halogen bonded, molecules. In a separate study we demonstrated that electron rich pyrimidines, related to molecule A, such as 5-(4-*N*,*N*-dimethylaminophenyl-ethynyl)pyrimidine, are viable ditopic halogen bond acceptors and form cocrystals with ditopic halogen bond donors such as 1,3-diiodotetrafluorobenzene. These cocrystals feature zig-zag one dimensional supramolecular polymers as shown in Figure 1b [13].

**Figure 2:**
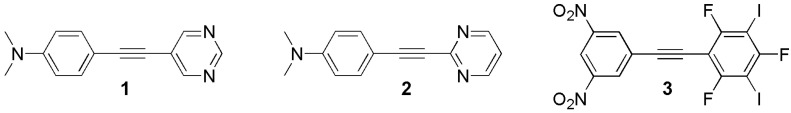
Compounds used in this study.

In this manuscript we incorporate ditopic halogen bonding of pyrimidines and π-π stacking. In particular we reasoned that *N*,*N*-dimethylaminophenylethynyl substituted pyrimidines **1** and **2** as both ditopic halogen bond acceptors and electron rich aromatics for EDA π-stacking when coupled with ditopic halogen bond donor 1-(3,5-dinitrophenylethynyl)-2,4,6-trifluoro-3,5-diiodobenzene, **3** (Figure 2) would form colored cocrystals. 

X-ray crystallography confirms the 3-dimensional arrangement of molecules within each of the cocrystals [**1•3**] and [**2•3**]. Indeed 1:1 cocrystals formed from separate equimolar solutions of the halogen bond donor **3** and each of the pyrimidines **1** and **2** feature 2 unique halogen bonds. They do, however, differ in the π-stacking arrangement within the crystals. Hirschfeld surface analysis using Crystal Explorer 17.5 [14] was used to evaluate close intermolecular contacts within the crystals as well as interaction energies between molecules within the crystalline structure. The relative strengths of individual halogen bonds and π-stacking interactions were calculated using the Gaussian16 software program (Wallingford, CT, USA) [15]. These calculations clearly show that EDA π-stacking is consistently stronger in these cocrystals than halogen bonding. Furthermore, separate calculation of the strengths of halogen bonding and π-stacking in the presence or absence of each other clearly demonstrate both the cooperative effect of halogen bonding on π-stacking and of π-stacking on halogen bonding. 

## 2. Results

### 2.1. Materials

**Figure 3:**
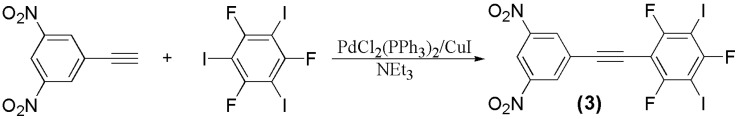
Synthesis of diiodotolane (**3**).

The compounds **1** and **2** were available from our previous study [13]. Sonogashira coupling of 1-iodo-3,5-dinitrobenzene with trimethylsilylacetylene followed by base deprotection yielded 3,5-dinitrophenylacetylene [12]. 3,5-Dinitrophenylacetylene was reacted with a 3-fold excess of 1,3,5-trifluoro-2,4,6-triiodobenzene to form the diiodo dinitrotolane (**3**) in 28 % yield shown in Figure 3.

The individual structures of the compounds **1** and **2** used in this study were confirmed by single crystal X-ray analysis and are included in the Supplementary Material.

### 2.2. Formation and Analysis of Cocrystals

Based on our expectation that the electron-rich pyrimidines **1** and **2** would each form 1:1 cocrystals with diiododinitrotolane **3** we dissolved the pairs of compounds in a 1:1 molar ratio in dichloromethane and allowed slow evaporation of the solvent. In each case the solutions become dark yellow brown as the volume of solvent decreased and ultimately bright red cocrystals formed.

**Figure 4:**
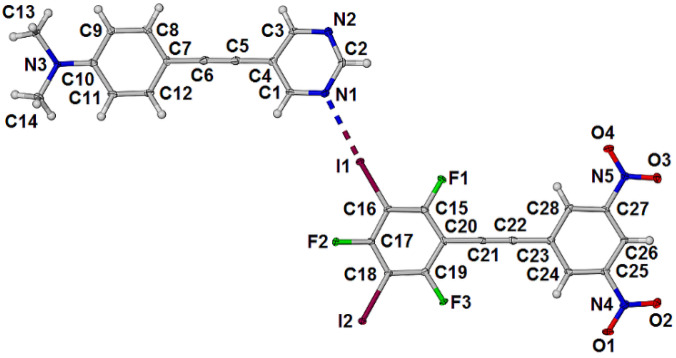
Asymmetric unit of the cocrystal **1•3** with displacement ellipsoids drawn at the 50% level.

The cocrystal **1•3** crystallized in the triclinic space group P-1. The asymmetric unit contains one molecule of each component as shown in Figure 4. There are two unique halogen bonds with distances I1∙∙∙N1 and I2#1∙∙∙N2 of 2.934(3) and 2.978(3) respectively and near linear angles C16-I1∙∙∙N1 and C18-I2∙∙∙N2#1 of 176.74(12) and 179.04(13)° respectively.

**Figure 5:**
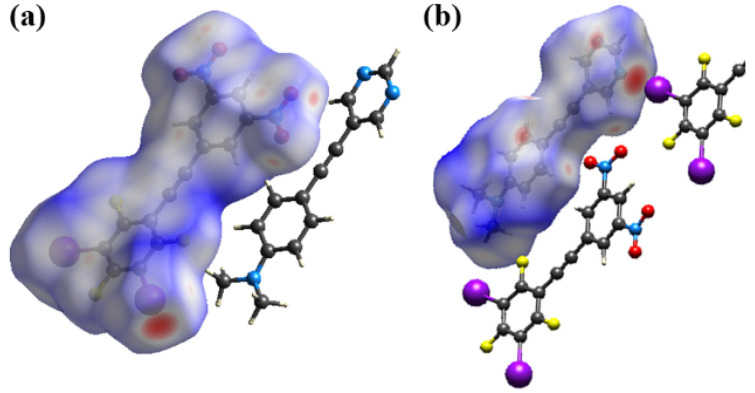
Hirschfeld surface showing *d_norm_* for **3** in (**a**) and for **1** in (**b**) highlighting the close contacts, in red, between adjacent molecules within the cocrystal **1•3**.

**Figure 6:**
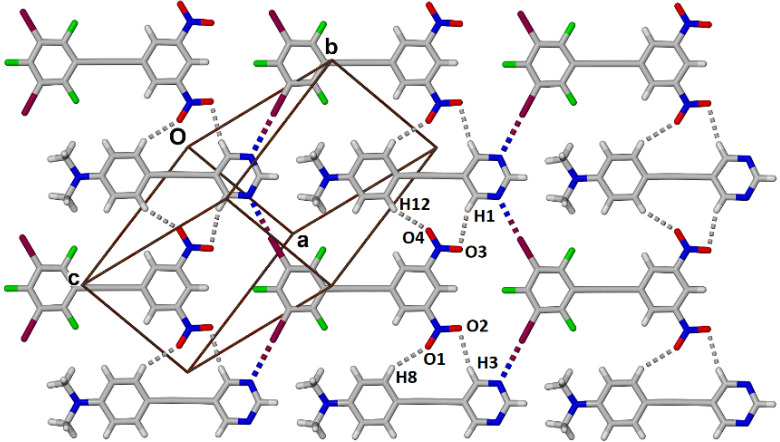
View of the planar sheet formed within the cocrystal **1•3** with halogen bonds shown as colored dashed lines and nonconventional hydrogen bonds shown as grey dashed lines.

The Hirschfeld surface was generated for each of the components and two complementary views are shown in Figure 5 and these surface plots confirm that the halogen bond is the most prominent close contact. These plots also reveal two bifurcated non-conventional C-H∙∙∙O contacts to each nitro group [16]. Thus, for nitro group O1N4O2 the H8∙∙∙O1 and H3∙∙∙O2 distances are 2.59 and 2.58 Å with C8-H8∙∙∙O1 and C3-H3∙∙∙O2 angles of 147.0 and 165.7° respectively (see Figure 6). For nitro group O3N5O4 the H1∙∙∙O3 and H12∙∙∙O4 distances are 2.55 and 2.58 Å with C12-H12∙∙∙O4 and C1-H1∙∙∙O3 angles of 149.0 and 159.5° respectively. In addition to these interactions within the plane of each molecule several less prominent close contacts resulting from π-stacking are visible on the Hirschfeld surface of each molecule.

Each of the two components, **1** and **3**, are essentially planar with an interplanar angle of 12.2(2)° between the planes of the pyridyl and dimethylaminophenyl rings and an interplanar angle of 5.9(3)° between the dinitrophenyl and perhalophenyl rings. Furthermore the interplanar angle across the halogen bond is 12.0(2)°. The halogen bonded interactions along with the cooperative C-H∙∙∙O interactions result in the formation of one-dimensional zig-zag supramolecular polymers that interdigitate to form an essentially planar 2-dimensional sheet shown in Figure 6.

**Figure 7:**
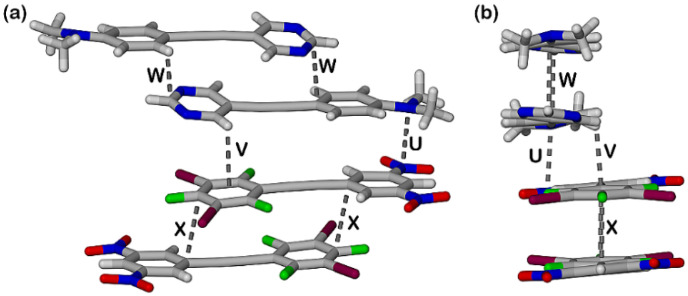
(**a**) Oblique view of the three unique stacking interactions **1-1**, **1-3** and **3-3** in the cocrystal **1•3** and view along the long axis of the four molecules in (**b**). Interactions U-X defined in the text.

The packing of the planar sheets is such that there are three unique offset π-stacking interactions. The expected π-stacked pair **1** and **3** is sandwiched between two offset head-to-tail π-dimers, **1-1** and **3-3** as shown in Figure 7. The π-stacked EDA complex, D-A in Figure 7, is sideways offset as evidenced by the dimethylamino-N atom being above one of the nitro nitrogen atoms with an N-N distance of 3.039(5) Å while H3 of the pyrimidine ring is located 3.17 Å above the centroid of the halogenated benzene. These interactions are labelled “U” and “V” in Figure 7. In contrast the **1-1** and **3-3** π-dimers are offset along the long axis of each molecule. Thus, two molecules of **1** are head-to-tail π-stacked with C2 of one molecule 3.162(6) Å above C7 of the second molecule, shown as “W” in Figure 7. In the π-stacking between two molecules of **3**, also stacked head-to-tail, C17 in the halogenated benzene of one molecule is 3.328(5) Å above C23 in the dinitrobenzene ring of the second molecule, “X” in Figure 7. Each of the interactions U to X are visible as red close contacts on the Hirschfeld surfaces shown in Figure 5.

**Figure 8:**
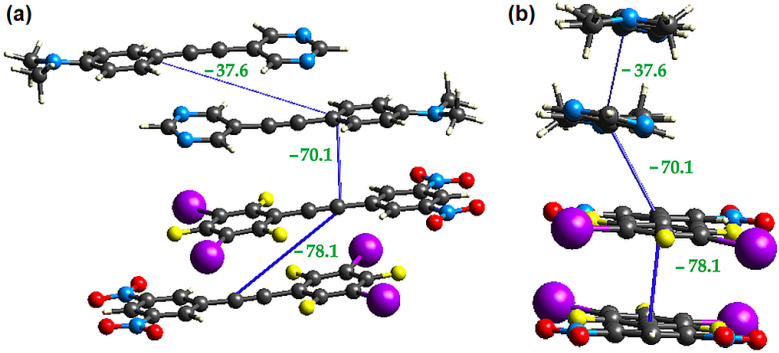
(**a**) Oblique view of the three unique stacking interactions **1-1**, **1-3** and **3-3** in the cocrystal **1•3** and view along the long axis of the four molecules in (**b**). Interactions’ energies (in kJ/mol) are in green.

To investigate the strength of the different π-stacking interactions present (**1-1**, **1-3** and **3-3**), interaction energies were calculated at the B3LYP/DGDZVP level of theory using Crystal Explorer 17.5. Crystal Explorer calculates the total interaction energies (E_total_) as a sum of electrostatic (E_ele_), polarization (E_pol_), dispersion (E_dis_) and exchange-repulsions terms (E_rep_). These calculations showed that the **3-3** π-dimer has the largest interaction energy, while the **1-1** π-dimer has the weakest, as seen in Figure 8. In all cases, the π-stacking interactions are driven mainly by dispersion and electrostatic interactions as shown in Table 1, with stacking interactions involving the large iodine atom having higher interaction energies due to large dispersion and electrostatic contributions. 

**Figure 9:**
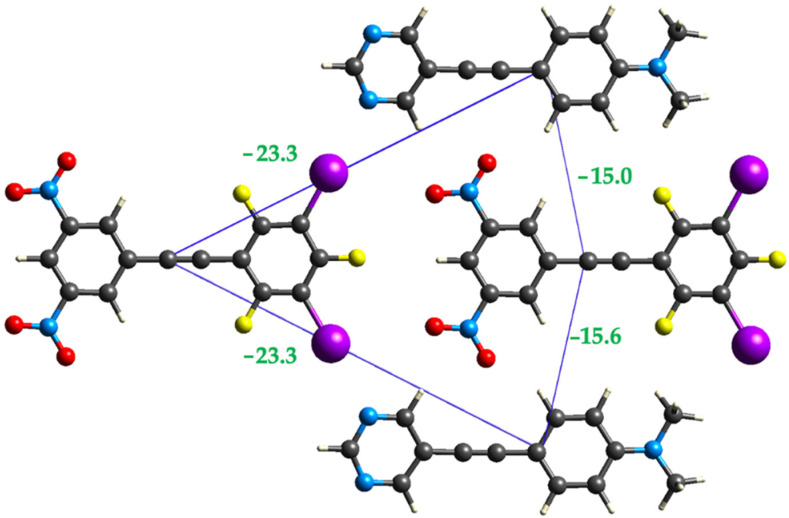
Crystal Explorer interaction energies (in kJ/mol) for halogen bonds and side-by-side interactions between halogen bond donor and acceptor.

We also examined the strength of the halogen bonds and non-conventional hydrogen bonds that occur between donor and acceptor molecules within the same plane of the cocrystal (Figure 9). These calculations show that the two halogen bonds are equivalent in strength, although examination of the individual components of the interaction energy shows some subtle differences in the individual components (Table 2) likely due to small differences in the intermolecular distances. The two side-by-side **1-3** interactions are quite similar in strength; however, we again observe some differences due to slightly different intermolecular distances between the two interacting species.

**Figure 10:**
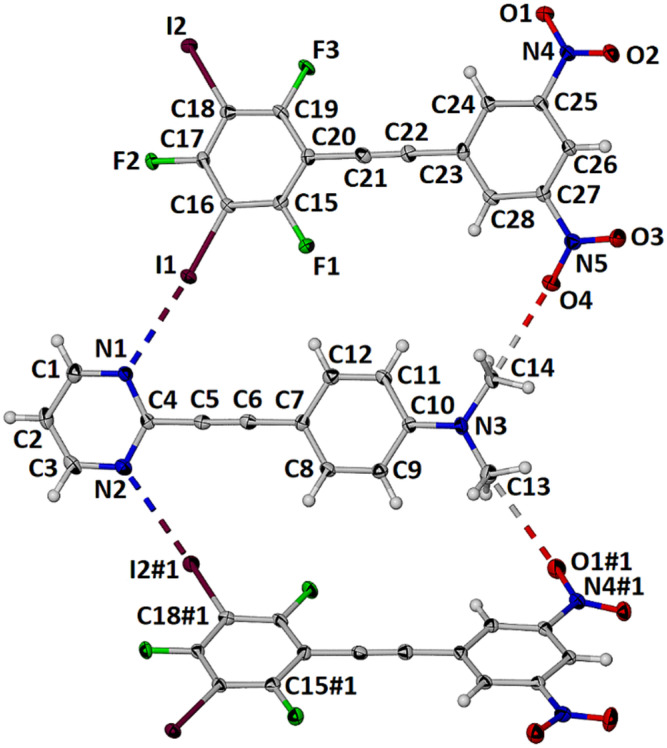
Asymmetric unit of the cocrystal **2•3** with displacement ellipsoids drawn at the 50% level. Halogen bonds and tetrel bonds shown as dashed lines.

The asymmetric unit of the cocrystal formed between **2** and **3** also included one molecule of each of the components as shown in Figure 10. Each of the component molecules **2** and **3** are essentially planar with interplanar angles of 5.84(14) and 8.08(12)° respectively between the aromatic rings in molecule **2** and **3**. A major difference to the **1•3** cocrystal is that the molecules do not form a planar sheet. Indeed the second diiodotrifluorophenyl ring has an interplanar angle of 46.92(7)° to the pyrimidine ring and C15#1 is 3.750(10) Å out of the plane defined by the pyrimidine ring (Figure 10). 

There are two unique halogen bonds with distances I1∙∙∙N1 and I2#1∙∙∙N2 of 3.022(3) and 3.261(3) respectively and angles C16-I1-N1 and C18#1-I2#1-N2 of 171.77(9) and 162.93(10)° respectively. These distances, while longer than those in the structure of **1•3**, are 86 and 92% of the sum of the van der Waals radii [17]. It is noteworthy that there are close interactions between nitro O atoms and the amino methyl C atoms. These interactions are reasonably described as tetrel interactions to C [18]. The C14∙∙∙O4 and C13#1∙∙∙O3 separations are 2.905(3) and 3.044(4) Å and the O4-C14-N3 and O1-C13#1-N3#1 angles are 177.5(2) and 165.5(2)° respectively. The C∙∙∙O separations are 89 and 94% of the sum of the van der Waals radii. These results are in accord with the statistical analysis of tetrel interactions between O and *sp*^3^-C bonded to N where more linear O-C-N angles correlated to shorter C∙∙∙O separations [19].

**Figure 11:**
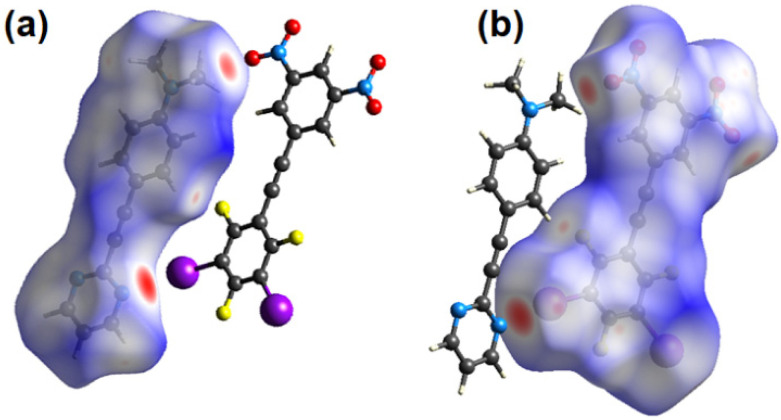
Hirschfeld surface showing *d_norm_* for **2** in (**a**) and for **3** in (**b**) highlighting the close contacts, in red, between adjacent molecules within the cocrystal **2•3**.

The Hirschfeld surfaces for each molecule shown in Figure 11 highlight these two major close contacts. Along with these two interactions there is a close C-H∙∙∙F contact with a H2∙∙∙F2 distance of 2.40 Å (92% the sum of the van der Waals radii). C-H∙∙∙F interactions are often observed in the structures of fluorinated benzenes [20]. 

**Figure 12:**
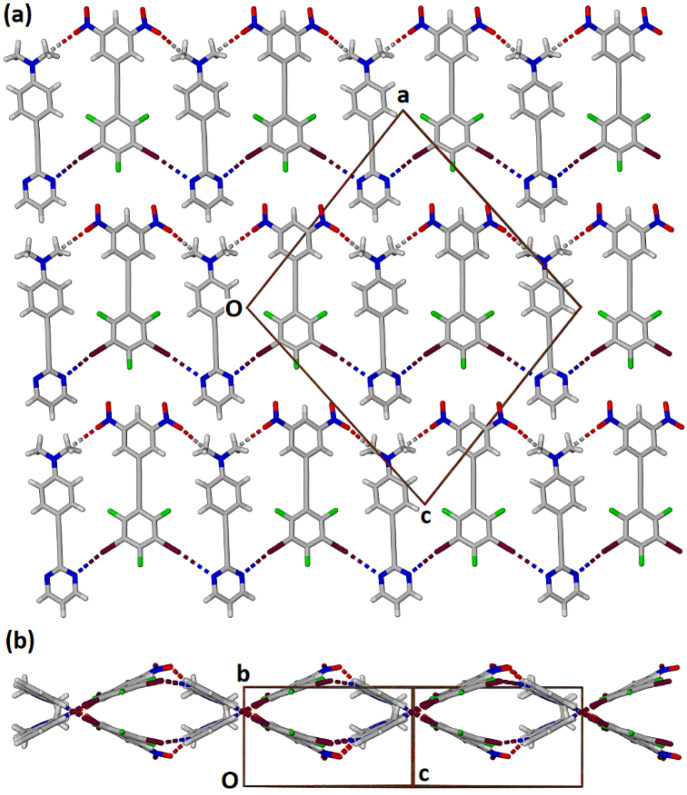
Two views of adjacent alternately corrugated halogen bonded ribbons in the cocrystal **2•3** with halogen bonds and tetral bonds shown as dashed lines. (**a**) View along the b axis and (**b**) view along the line 1 0 −1.

In contrast to the structure **1•3** the one-dimensional ribbons of halogen bonded molecules in **2•3** are alternately corrugated as shown in Figure 12.

**Figure 13:**
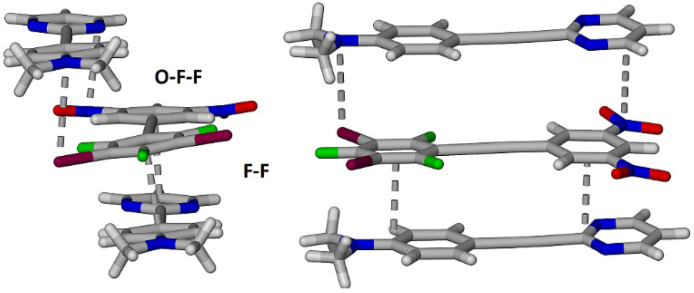
Complementary views of the two unique π-stacking interactions between three adjacent molecules within the cocrystal **2•3**.

In contrast to the complex π-stacking observed in **1•3**, the molecules within the cocrystal **2•3** do stack in alternate of **2** and **3**. However, the stacking is alternately offset along the short axis of each molecule as shown in Figure 13. The face-to-face **2-3** orientation, F-F in Figure 13, is also different with the pyrimidine ring and the dinitrophenyl ring stacked rather than the expected stacking of the *N*,*N*-dimethylaminophenyl ring stacked with the dinitrophenyl ring as in cocrystal **1•3**. Thus, in the face-to-face π-interaction the centroid of the dinitrophenyl lies almost directly above the ipso carbon on the pyrimidine ring. The second, offset, π-stacking interaction, O-F-F in Figure 13, has the dimethylamino N atom aligned with one of the iodine atoms on the diiodotrifluorophenyl ring.

**Figure 14:**
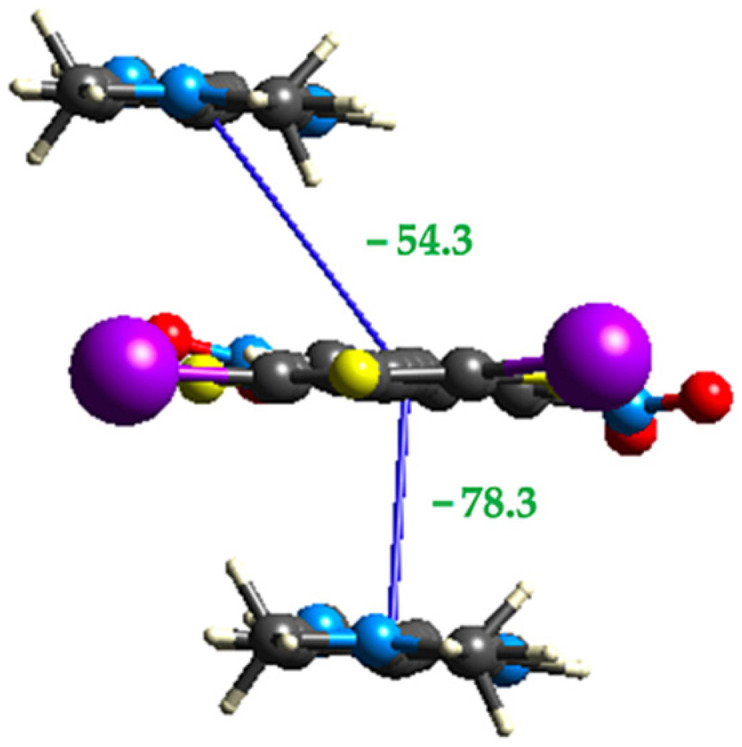
Crystal Explorer interaction energies (in kJ/mol) for the two π-stacking interactions within the cocrystal **2•3**.

Crystal Explorer calculations examining the interaction energies for the two π-stacked dimers show that the offset interaction is weaker than the face-to-face interaction (Figure 14) due to a significant decrease in both the electrostatic and dispersion contributions (Table 3).

**Figure 15:**
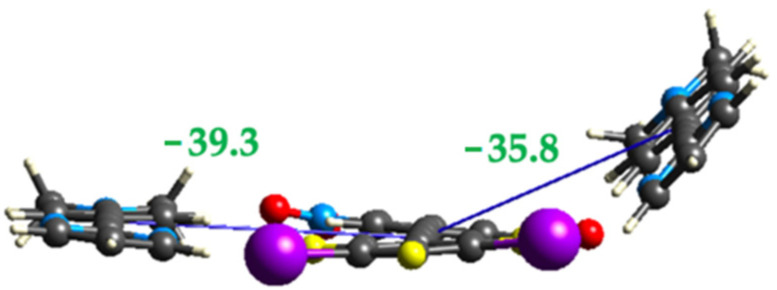
Crystal Explorer interaction energies (in kJ/mol) for the two halogen bonding interactions within cocrystal **2•3**.

As expected, calculations on the two halogen bonds show that the halogen bond strength decreases as the halogen bond length increases and the C-I-N angle deviates farther from 180 (Figure 15). Because the halogen bonding and side-by-side interactions in **1•3** stem from different partners, these can be considered separately (Table 2). In **2•3**, however, the side-by-side nature of the halogen bonding partners precludes similar treatment. In effect, the side-by-side E_totals_ for **2•3** encompass both the halogen bonding and the tetral bonding that is absent in **1•3** (Table 4). Consequently, the interaction energies between halogen bonded molecules in the cocrystal **2•3** are significantly higher than those in cocrystal **1•3**. This is likely due to the added contribution of the tetral bond in cocrystal **2•3**.

### 2.3. Computational Analysis of the Interplay between Halogen Bonding and π-Stacking

To evaluate the interplay between halogen bonding and π-stacking and whether the interactions show any cooperativity, trimers that featured a monomer involved in both halogen bonding and π-stacking were taken from the crystal structure and subjected to electronic structure calculations. For each trimer, the strength of the interactions was calculated within the trimer, and as an isolated dimer, and compared. For cocrystal **1•3** this is complicated since there are three unique π-stacking interactions that are treated separately.

**Figure 16:**
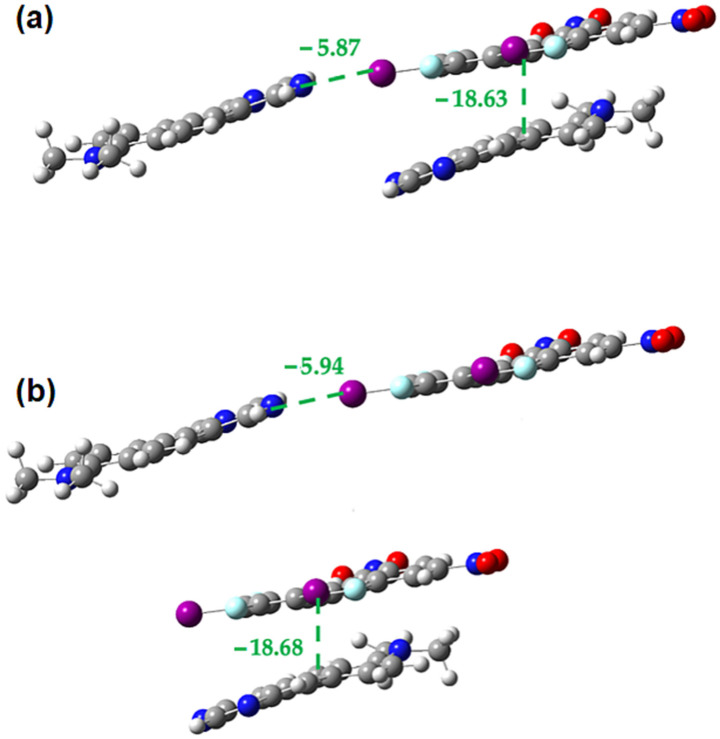
M062X-D3/DGDZVP interaction energies (in kcal/mol) in the **1•3** cocrystal for the interplay between **1-3** π-stacking and halogen bonding (**a**) within trimer (**b**) in the isolated dimers.

Figure 16 shows that when a halogen bond donor in the **1•3** cocrystal is involved in both an electron donor-acceptor π-interaction and a halogen bond there is no significant cooperativity or competition between the two. While small differences in energy are noted, they are possibly due to inaccuracies in the computational methods rather than any true cooperativity/competition. Calculations (results not shown) in which the halogen bond acceptor of the **1-3** π-dimer is making a halogen bond give the same result. 

**Figure 17:**
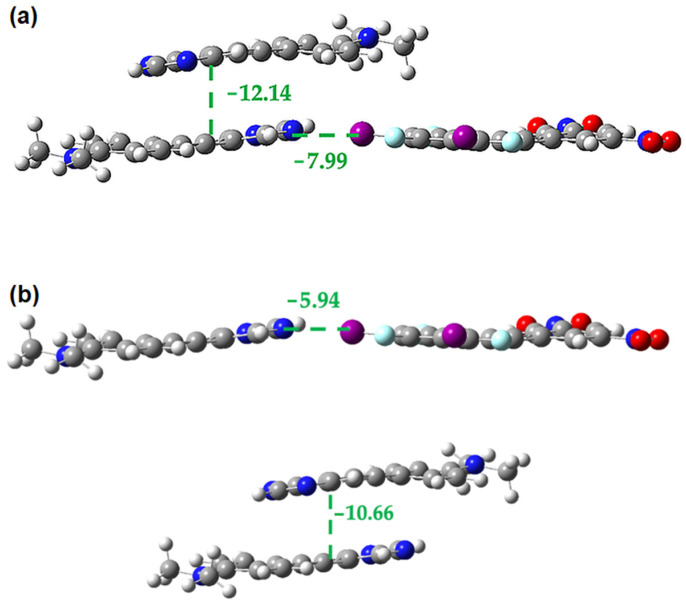
M062X-D3/DGDZVP interaction energies (in kcal/mol) in the **1•3** cocrystal for the interplay between **1-1** π-stacking and halogen bonding (**a**) within trimer (**b**) in the isolated dimers.

**Figure 18:**
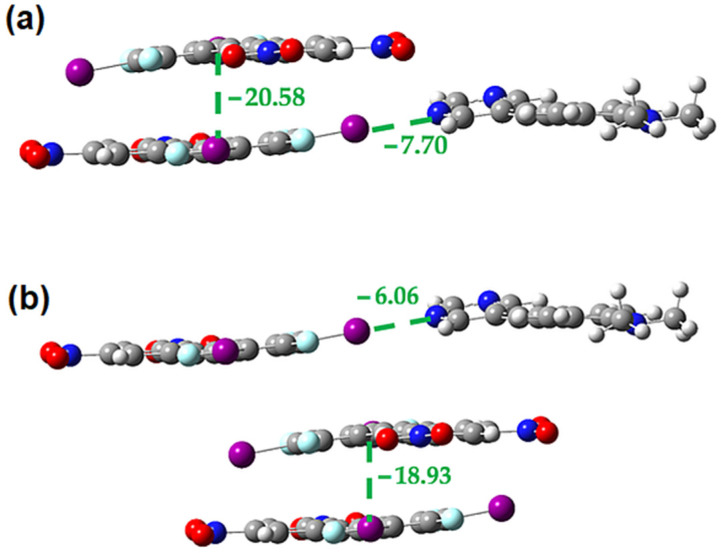
M062X-D3/DGDZVP interaction energies (in kcal/mol) in the **1•3** cocrystal for the interplay between **3-3** π-stacking and halogen bonding (**a**) within trimer (**b**) in the isolated dimers.

Figure 17 and Figure 18 show the interplay between the **1-1** π-interaction and the halogen bond and the **3-3** π-interaction and the halogen bond, respectively. In both cases we see that the strength of these interactions increases by ~2 kcal/mol in the presence of one another, indicating that the two are mutually cooperative. 

**Figure 19:**
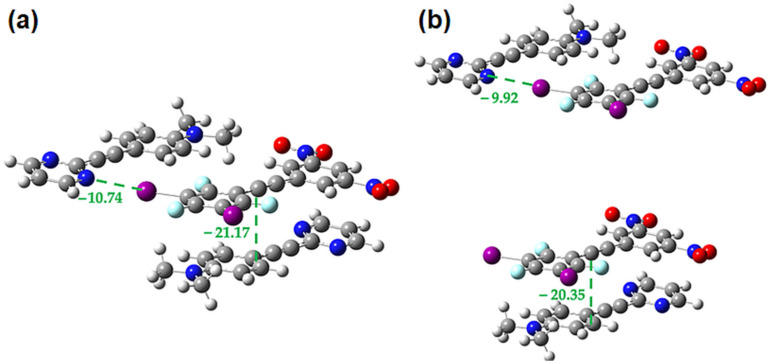
M062X-D3/DGDZVP interaction energies (in kcal/mol) in the **2•3** cocrystal for the interplay between the face-to-face **2-3** π-stacking and the linear halogen bond (**a**) within trimer (**b**) in the isolated dimers.

**Figure 20:**
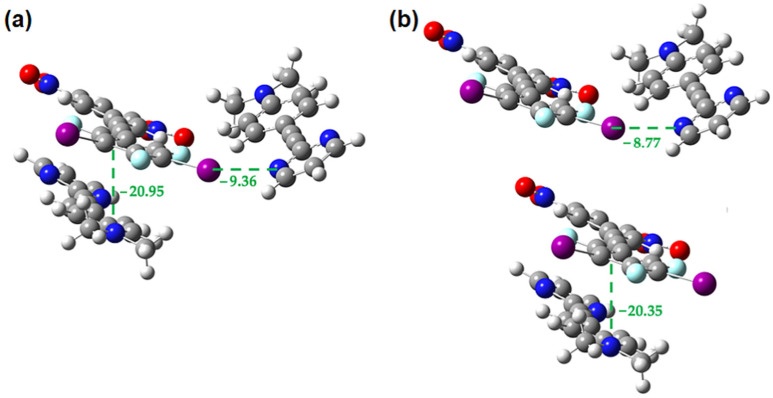
M062X-D3/DGDZVP interaction energies (in kcal/mol) in the **2•3** cocrystal for the interplay between the face-to-face **2-3** π-stacking and the bent halogen bond (**a**) within trimer (**b**) in the isolated dimers.

A similar analysis was carried out on the **2•3** cocrystal. Figure 19 and Figure 20 suggest that there may be weak cooperativity between the face-to-face π-stacking interaction and the halogen bonds since in both cases the strength of both interactions increase slightly in the trimer, relative to the isolated dimers. The magnitude of the difference (~0.6 kcal/mol) is like previous work [21,22] and may indicate weak cooperativity, however, the difference is small enough that the evidence is not conclusive.

**Figure 21:**
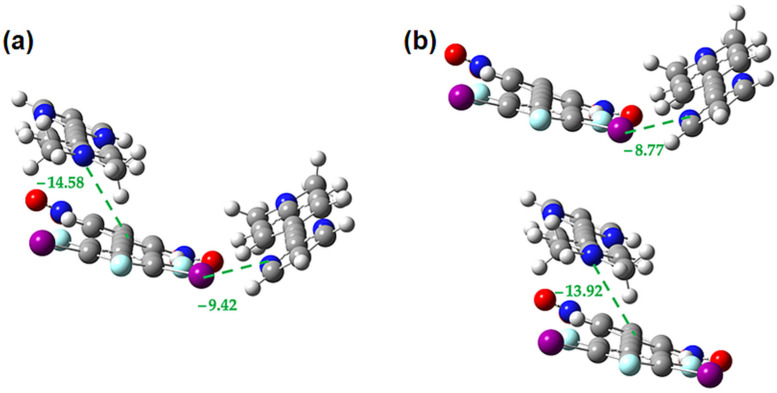
M062X-D3/DGDZVP interaction energies (in kcal/mol) in the **2•3** cocrystal for the interplay between the offset **2-3** π-stacking and the bent halogen bond (**a**) within trimer (**b**) in the isolated dimers.

**Figure 22:**
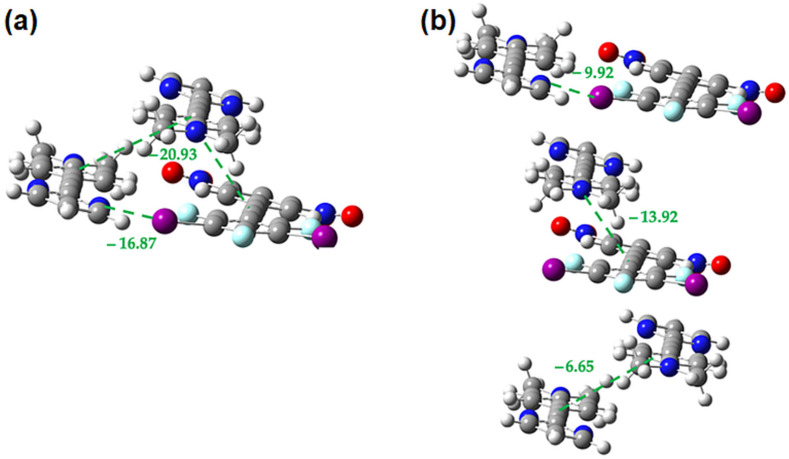
M062X-D3/DGDZVP interaction energies (in kcal/mol) in the **2•3** cocrystal for the interplay between the offset **2-3** π-stacking and the linear halogen bond (**a**) within trimer (**b**) in the isolated dimers.

Analysis of the interplay between the offset **2-3** π-stacking interaction and the bent halogen bond shows a similar result to that of the face-to-face **2-3** π-stacking (Figure 21). When considering the interplay between the offset π-stacking interaction and the linear halogen bond, however, the analysis is complicated by the presence of a non-negligible interaction between the two acceptors (Figure 22). However, if we compare the strength of the π-stacking interaction in the trimer (−20.93 kcal/mol) to the sum of the energies for the two π-stacking dimers (−20.57 kcal/mol) we see that they are very close, indicating no cooperativity. Similarly, if we compare the halogen bond energy in the trimer (−16.87 kcal/mol) to the sum of the energy to break the halogen bond and the stacking interaction in the isolated dimers (−16.57 kcal/mol) we again see that the values are quite close indicating that there is little to no cooperativity.

## 3. Materials and Methods

### 3.1. Preparation of Materials

Compounds **1** and **2** were available from a previous study [11]. The single crystal X-ray structure of **1** is included in the supporting information. The structure of **2** has been published [23,24]. Compound **3** was prepared through Sonogashira coupling of the corresponding iodoarene and terminal alkyne as shown in Figure 3.

Synthesis of 1,3,5-Triflouro-2,4-diiodo-(3,5-dinitrophenylethynyl)benzene, **3**. 1-Ethynyl-3,5-dinitrobenzene (0.353 g, 1.83 mmol) and 1,3,5-trifluoro-2,4,6-triodobenzene (2.763 g, 5.42 mmol) were dissolved in a mixture of triethylamine (2 mL) and tetrahydrofuran (5 mL) and argon bubbled through the mixture for 5 min. Bis(triphenylphosphine) palladium(II) chloride (0.035 g) and copper iodide (0.017 g) were added and the reaction mixture was heated at 45 °C for 12 h under an argon atmosphere. The reaction was cooled to room temperature and solvent removed in vacuo. The crude product was diluted with dichloromethane and washed with water, followed by brine. After evaporation of the solvent, the crude material was dry loaded onto a silica column and purified by flash column chromatography. Hexanes was run first to recover unreacted 1,3,5-trifluoro-2,4,6-triodobenzene and then the column eluted with increasingly polar mixtures of hexanes/EtOAc until the title compound eluted with 15:1 hexanes:ethyl acetate mixture as colourless crystals (0.285 g, 27%. ^1^H NMR (400 MHz): δ 9.08 (1H, t, *J* 2.3 Hz), 8.73 (2H, d, *J* 2.3 Hz). ^19^F NMR (400 MHz): δ −84.1 (d, *J* 4.0 Hz, 2F), −64.3 (t, *J* 4.0 Hz, 1F).

### 3.2. Cocrystallization

5-(4-*N*,*N*-dimethylaminophenylethynyl)pyrimidine, **1**, (2.6 mg, 0.013 mmol) and 1,3,5-triflouro-2,4-diiodo-(3,5-dinitrophenylethynyl)benzene, **3** (7.4 mg, 0.013 mmol) were weighed into a screw cap vial. Dichloromethane (2 mL) was added and the mixture vortexed until a homogeneous solution was obtained. The solvent was allowed to slowly evaporate until homogeneous mass of clear red cocrystals, **1•3**, formed. Cocrystal **2•3** was formed following a similar procedure.

### 3.3. Structure Refinement 

Crystal data, data collection and structure refinement details for cocrystals **1•3**, **2•3** and pyrimidines **1** and **2** are summarized in Table S1. Single crystals of each were mounted on a Kryoloop using viscous hydrocarbon oil. Data were collected at 100 K using a Bruker Apex1 CCD diffractometer equipped with Mo Kα radiation with λ = 0.71073 Å. Low temperature data collection was facilitated by use of a Kryoflex system with an accuracy of ±1 K. Initial data processing was carried out using the Apex 2 software suite (Madison, WI, USA) [25]. Structures were solved by direct methods using SHELXT-2018 [26] and refined against F2 using SHELXL-2018 [27]. The program X-Seed was used as a graphical interface [28]. The aromatic H atoms, all observed in the difference maps, were treated as riding atoms in geometrically idealized positions with C—H = 0.95 (aromatic) or 0.98 Å (methyl) and Uiso (H) = kUeq (C). 

### 3.4. Computational Methods 

Interaction energies for the crystal were calculated using Crystal Explorer 17.5 [14,29] at the B3LYP/DGDZVP level of theory [30,31,32,33]. The cooperativity and competition between the different intermolecular interactions was explored by carrying out single point energy calculations on the relevant clusters from the crystal structure using the Gaussian16 (rev B.01) software program [15]. All energy calculations were carried out using the M062X-D3 level of theory [34,35] with the DGDZVP basis set [32,33], as used in previous work [12]. All interaction energies were calculated relative to the unrelaxed monomers and were counterpoise corrected [36,37] for basis set superposition error.

## 4. Conclusions

In conclusion, this combined experimental and computational study demonstrates successful application of cooperative ditopic halogen bonding and electron donor-acceptor π-stacking to the formation of highly colored cocrystals. Importantly, the halogen bonding and π-stacking are both complementary and cooperative.

## Figures and Tables

**Table 1:** Interaction energies (in kJ/mol) for the unique stacking interactions in the the cocrystal **1•3**.

π-Dimer	E_ele_	E_pol_	E_dis_	E_rep_	E_total_
D-D	−53.0	−3.4	−109.9	123.3	−78.1
D-A	−48.9	−4.8	−88.3	100.6	−70.1
A-A	−34.7	−2.3	−59.0	84.4	−37.6

**Table 2:** Intermolecular distances (in Å) and interaction energies (in kJ/mol) for the halogen bonded molecules and the D-A side-by-side interactions in cocrystal **1•3**.

Interaction Type	R ^1^	E_ele_	E_pol_	E_dis_	E_rep_	E_total_
Halogen bond	12.21	−46.8	−5.6	−9.5	62.5	−23.3
Halogen bond	12.25	−42.3	−5.1	−9.3	53.9	−23.3
Side-by-side	8.41	−13.3	−2.5	−27.2	39.8	−15.0
Side-by-side	8.44	−11.5	−2.5	−26.0	34.0	−15.6

^1^ R is the distance between the molecular centroids.

**Table 3:** Interaction energies (in kJ/mol) for the unique stacking interactions in cocrystal **2•3**.

π-Dimer	E_ele_	E_pol_	E_dis_	E_rep_	E_total_
O-F-F	−35.5	−5.2	−72.9	81.9	−54.3
F-F	−54.7	−6.5	−96.7	110.0	−78.4

**Table 4:** C-I-N angles and interaction energies (in kJ/mol) between halogen bonded molecules in cocrystal **2•3**.

**r_XB_** **(Å)**	**C-I-N Angle** **(Degrees)**	**r_TB_** **(Å)**	**E_ele_**	**E_pol_**	**E_dis_**	**E_rep_**	**E_total_**
3.022(3)	171.77(9)	2.905(3)	−49.9	−7.5	−27.3	69.4	−39.3
3.261(3)	162.93(10)	3.044(4)	−34.3	−5.3	−31.4	51.3	−35.8

## Data Availability

Crystallographic data has been deposited with the CCDC.

## Supplementary Materials

The following are available online, Table S1, Table S2 and Figure S1: Crystallographic data.

## Sample Availability

Samples of the compounds are not available from the authors.
