# Supplementary material for: Complementary, Cooperative Ditopic Halogen Bonding and Electron Donor-Acceptor π-π Complexation in the Formation of Cocrystals
Source: Molecules. 2022 Feb 24;27(5):1527. doi: 10.3390/molecules27051527 (PMC8911696; doi:10.3390/molecules27051527)
Supplement: Supplementary file 1 [file molecules-27-01527-s001.zip › molecules-1551716-supplementary.pdf]

Supporting information

Cooperative Ditopic Halogen Bonding and Electron Donor-Acceptor π–π Complexation in the Formation of Cocrystals

Erin D. Speetzen [1,*], Chideraa Nwachukwu [2], Nathan P. Bowling [1], and Eric Bosch [2,*]

[1] Department of Chemistry, University of Wisconsin-Stevens Point, 2001 South Avenue, Stevens Point, WI 54481, USA
[2] Chemistry Department, Missouri State University, 901 South National Avenue, Springfield, MI 65897, USA
* Correspondence: ericbosch@missouristate.edu

Table of Contents

Table S1. Crystallographic data and selected data collection parameters 2

Table S2. Halogen and tetral bond distances and angles..3

Figure S1. Asymmetric unit of the structure **1** with displacement ellipsoids drawn at the 50% level...4

Table S1. Crystallographic data and selected data collection parameters.

**Cocrystal**	**1•3**	**2•3**	**1**	**2**
empirical formula	$C_{14}H_3F_3I_2N_2O_4$, $C_{14}H_{13}N_3$	$C_{14}H_3F_3I_2N_2O_4$, $C_{14}H_{13}N_3$	$C_{14}H_{13}N_3$	$C_{14}H_{13}N_3$
crystal system	triclinic	monoclinic	monoclinic	orthorhombic
space group	*P-1*	*P21/n*	*P21*	*Pna21*
a (Å)	10.4746(12)	19.1046(12)	6.1671(5)	11.8950(5)
b (Å)	11.5979(13)	7.4025(5)	26.405(2)	9.0010(4)
c (Å)	13.0950(15)	20.1452(13)	7.4662(7)	c=11.0201(5)
α (°)	111.104(2)	90	90	90
β (°)	109.936(2)	99.5310(10)	95.6870(10)	90
γ (°)	94.287(2)	90	90	90
Volume ($Å^3$)	1359.3(3)	2809.6(3)	1209.82(18)	1179.89(9)
Z	2	4	4	4
Dc ($g \cdot cm^{-3}$)	1.948	1.885	1.226	1.257
μ (mm^{-1})	2.380	2.303	0.0705	0.077
No. of ref. total	17353	34692	15969	14574
No. of ref. unique	4991	5333	5422	2503
No. of param.	381	381	312	156
R1	0.0333	0.0364	0.0404	0.0353
*R1*all	0.0426	0.0294	0.0449	0.0372
wR2	0.0731	0.0675	0.1048	0.0907
w*R2*all	0.0784	0.0709	0.1082	0.0923
GooF	1.026	1.066	1.065	1.062
CCDC #	2108080	2108081	2129574	2129575

Table S2. Halogen bond and tetrel bond distances and angles.

Structure	N---I (Å)	N-I-C (°)	O---C(Å)	O-C-C (°)
**1•3**	2.934(3)	176.74(12)		
	2.978(3)	179.04(13)		
**2•3**	3.022(3)	171.77(9)	2.905(3)	177.5(2)
	3.261(3)	162.93(10)	3.044(4)	165.5(2)

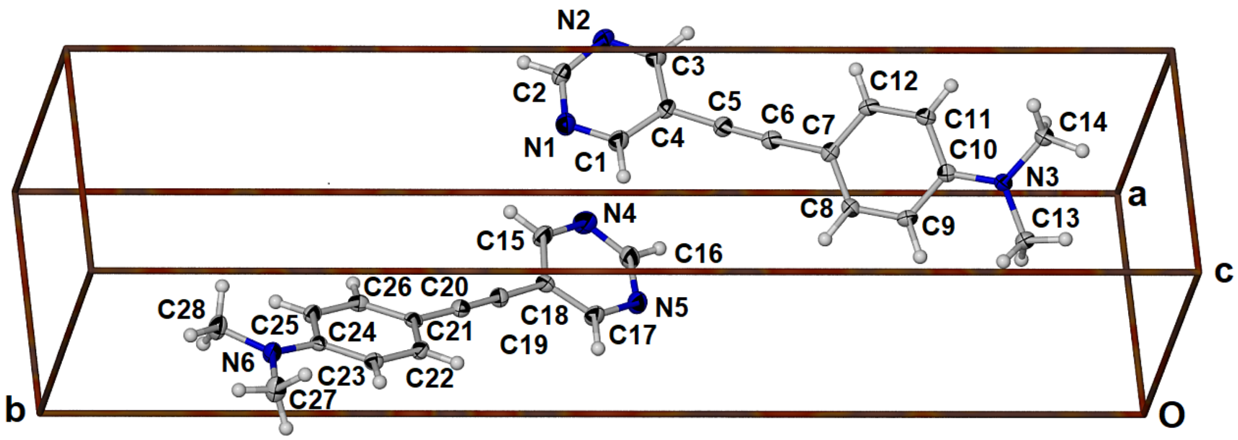

Figure S1. Asymmetric unit of the X-ray structure of **1** showing both unique molecules with the displacement ellipsoids drawn at the 50% level.
